# Progress in reducing premature mortality from cancer and cardiovascular disease in the former Soviet Union, 2000–19

**DOI:** 10.1093/eurpub/ckac030

**Published:** 2022-04-20

**Authors:** Ariana Znaor, Marilys Corbex, Bochen Cao, Mathieu Laversanne, Anton Ryzhov, Vitaly Smelov, Freddie Bray

**Affiliations:** Cancer Surveillance Branch, International Agency for Research on Cancer, Lyon, France; Division of Country Health Programs, World Health Organization Regional Office for Europe, Copenhagen, Denmark; Division of Data, Analytics and Delivery for Impact, World Health Organization, Geneva, Switzerland; Cancer Surveillance Branch, International Agency for Research on Cancer, Lyon, France; Department of General Mathematics, Taras Shevchenko National University of Kyiv, Kyiv, Ukraine; Division of Country Health Programs, World Health Organization Regional Office for Europe, Copenhagen, Denmark; Cancer Surveillance Branch, International Agency for Research on Cancer, Lyon, France

## Abstract

**Background:**

A reduction in non-communicable diseases premature mortality by one-third by 2030 is one of the targets of the UN Sustainable Development Goals (SDG3.4). We examined the mortality profiles in the Newly Independent States of the former Soviet Union (NIS) and the European Union (EU) and assessed progress in reductions of premature mortality from cancer, as compared to cardiovascular disease (CVD).

**Methods:**

We used WHO’s Global Health Estimates and GLOBOCAN 2020 to examine current mortality profiles and computed the unconditional probabilities of dying at ages 30–70 from CVD and cancer for the years 2000–19 in both sexes, using a linear extrapolation of this trend to predict whether the target of a one-third reduction, as set in 2015, would be met in 2030.

**Results:**

CVD was the main cause of premature death in the NIS (43%), followed by cancer (23%), inversely from the EU with 42% cancer and 24% CVD deaths. The NIS achieved major reductions in premature CVD mortality, although the probabilities of death in 2019 remained about five times higher in the NIS compared to the EU. For cancer, mortality reductions in most NIS were quite modest, other than large declines seen in Kazakhstan (44%) and Kyrgyzstan (30%), with both on course to meet the 2030 target.

**Conclusions:**

Limited progress in cancer control in the NIS calls for policy action both in terms of structural changes towards universal health coverage, and scaling up of national cancer control plans, including a shift from opportunistic to evidence-based early detection practices.

## Introduction

In 2015, all UN Member States adopted the 2030 Agenda for Sustainable Development, comprising 17 Sustainable Development Goals (SDG) and 163 targets; the SDG target 3.4 is a reduction in non-communicable disease (NCD) premature mortality (ages 30–70) by one-third between 2015 and 2030.^1^

The European Region of the World Health Organization (WHO/Europe) consists of 53 countries with over 1.2 billion people, including a unique economic and political European Union (EU) between 27 EU countries, and the countries commonly referred to as the Newly Independent States of the Soviet Union (NIS).^2–4^ While premature mortality from NCDs in WHO/Europe has declined steadily since the mid-1990s, the magnitude and trends of premature mortality vary widely across sub-regions and countries, and the NIS are among the countries with highest burden from NCDs, with a marked excess mortality relative to countries within the EU.^5^^,^^6^

With cancer and cardiovascular disease (CVD) responsible for two-thirds of all premature deaths,^5^ the aim of this study was to examine the current premature mortality landscape in the NIS vs. the EU, and for selected countries, compare national trends in the probabilities of death from CVD and cancer, assessing whether a one-third reduction in premature mortality from cancer will be realized by 2030.

## Methods

NIS comprised the following 12 countries: Armenia, Azerbaijan, Belarus, Georgia, Kazakhstan, Kyrgyzstan, Republic of Moldova, the Russian Federation, Tajikistan, Turkmenistan, Ukraine and Uzbekistan; this excluded the Baltic countries (Estonia, Latvia and Lithuania) that form a part of the EU since 2004. We also defined the EU-27 + 1 as the former EU-28, namely the EU-27 countries plus the UK, consistently to the analyzed data period.

We used the GLOBOCAN 2020 estimates to examine the scale and profile of cancer mortality at ages 30–70 in these two regions in 2020.^7^ The details on methodology and data sources for deriving the estimates have been described elsewhere;^8^ they are available online at IARC’s Global Cancer Observatory website.^7^

The WHO’s Global Health Estimates (GHE) were used to estimate the main causes of death in the NIS and in the EU-27 + 1 in 2019 and assess recent trends in the 12 NIS and 12 selected comparison countries from EU-27 + 1.^5^ We computed the unconditional probabilities of dying between ages 30 and 70 years from CVD and cancer for the years 2000–19 in both sexes. A life table method allows calculation of the risk of death between exact ages 30 and 70 from CVD or cancer, using age-specific mortality rates from GHE.^5^ The method is described in detail in NCD Global Monitoring Framework: Indicator Definitions and Specifications.^9^

To assess the change in trends for cancer set against the SDG target 3.4, we calculated the percentage change in the probability of premature deaths from cancer in 2000–19. A linear extrapolation of this trend was then used to predict whether the national target of a one-third reduction between the year 2015 and 2030 would likely be met. GLOBOCAN 2020 and GHE data are available by 5-year age groups, for ease of interpretation and consistency with the SDG target 3.4, we describe the age-group 30–69 as ‘30-70’.

## Results

### Premature mortality profiles

**Figure 1:**
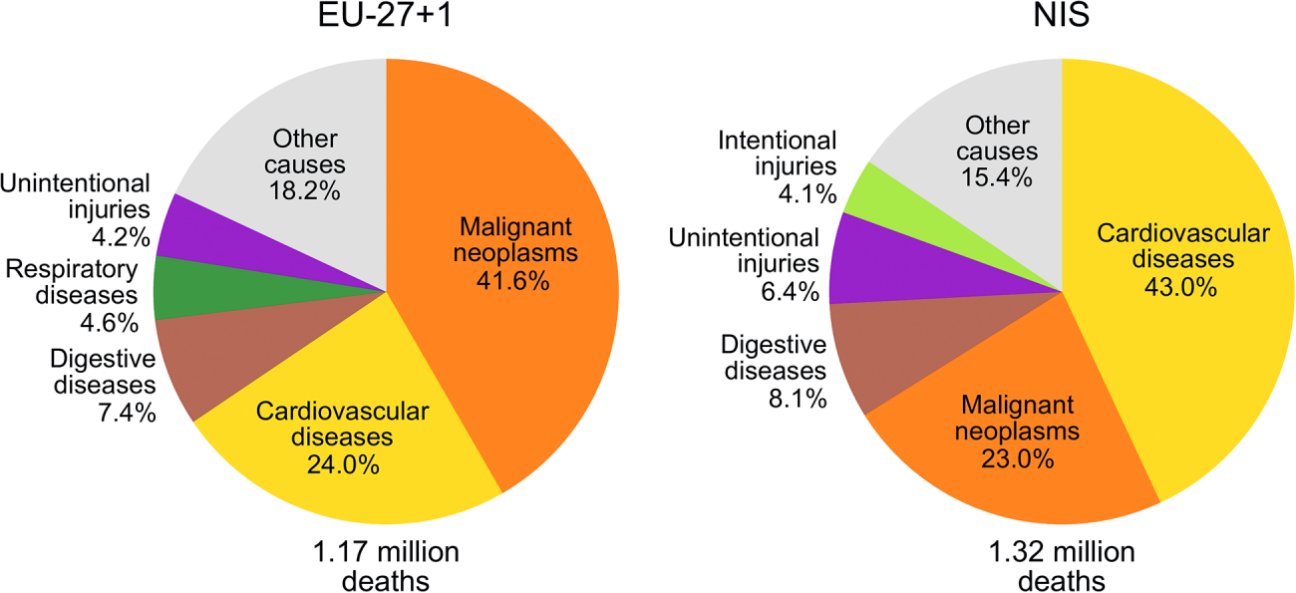
Five most common causes of premature death in the NIS and EU-27 + 1 in 2019, ages 30–70. EU-27 + 1, 27 EU countries and the UK; NIS, Newly Independent States of the former Soviet Union

The profiles of premature mortality differ between the EU-27 + 1 and the NIS (figure 1). Cancer was the leading cause of premature death in EU-27 + 1 in 2019 (41.6% deaths at ages 30–70) with CVD contributing to 24.0% premature deaths, while in the NIS, the leading causes were CVD (43.0%), with cancer responsible for 23.0% of premature deaths. In both regions, proportions of cancer deaths were higher, and proportions of CVD deaths lower in women than in men (Supplementary figure S1A and B).

**Figure 2:**
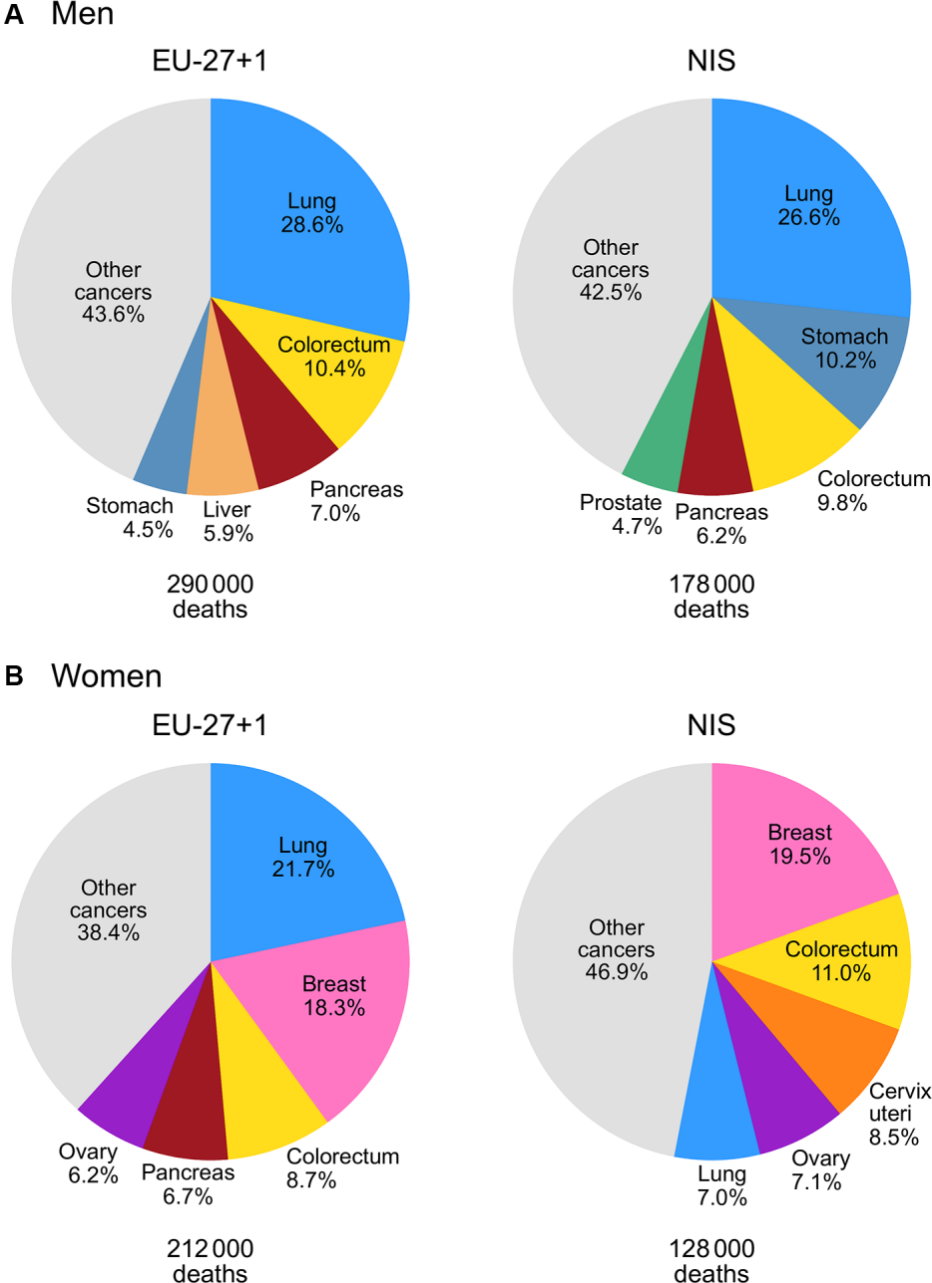
Five most common causes of premature cancer death in the NIS and EU-27 + 1 in 2019, ages 30–70, men (A) and women (B). EU-27 + 1, 27 EU countries and the UK; NIS, Newly Independent States of the former Soviet Union

Figure 2 presents the most common causes of premature cancer mortality in the EU-27 + 1 and the NIS. In men, lung cancer was the most common cause of premature cancer death in both sub-regions, contributing to over one-quarter of all premature cancer deaths in each sub-region. Stomach cancer was second most common in the NIS (10.2%) but ranked fifth in the EU-27 + 1 (4.5%). Colorectal cancer ranked as the second leading cause of premature death in men in EU-27 + 1 (10.4%) and third in the NIS (9.8%).

The most common cause of premature cancer death in women in EU-27 + 1 was lung cancer (21.7% of female cancer deaths), the fifth most frequent cause of cancer death in women in the NIS. Cervical cancer ranked third in women in NIS (8.5%), and sixth in the EU-27 + 1 (4.0%). Stomach cancer was the sixth most common cause of female cancer deaths in the NIS (6.7%), while not among the top 10 causes of premature cancer death in the EU-27 + 1.

### Trends in CVD and cancer premature deaths 2000–19

**Figure 3:**
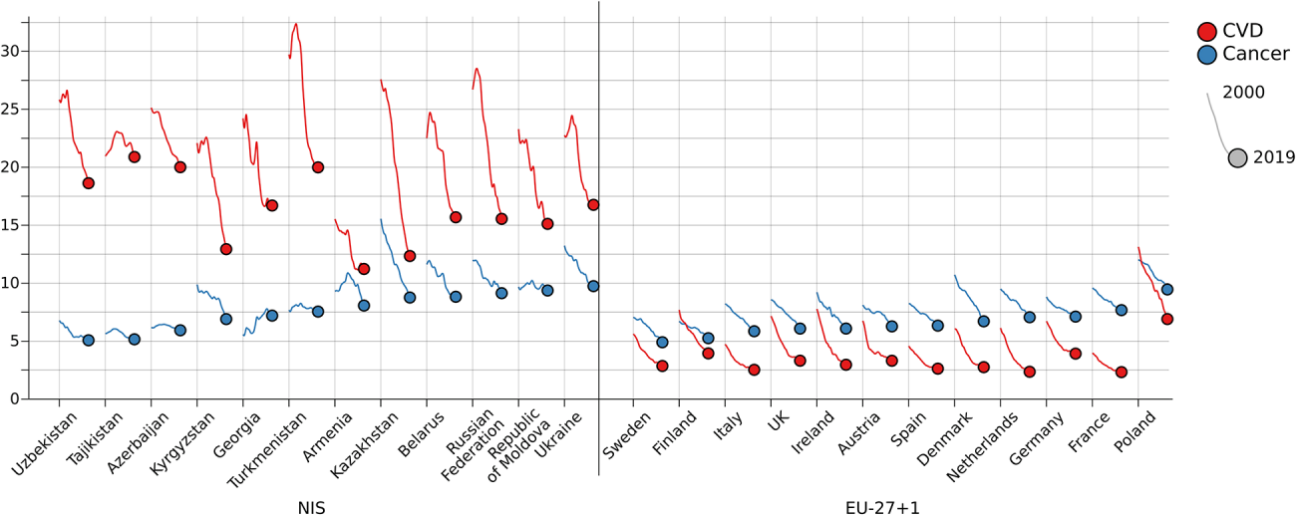
Trends in the probability of premature death (ages 30–70) in NIS vs. selected EU-27 + 1 countries, 2000–19. EU-27 + 1, 27 EU countries and the UK; NIS, Newly Independent States of the former Soviet Union; Probability of dying, unconditional probability of dying between ages 30 and 70 years; CVD, cardiovascular disease

Figure 3 shows trends in the probability of premature death (ages 30–70) from 2000 to 2019 in selected NIS vs. selected EU-27 + 1 countries. In the year 2000, there were large variations in the probabilities of dying from CVD and cancer within the NIS. In each country, the probability of dying from CVD was 2- to 4-fold higher than from cancer. In the EU countries, the probability of dying from CVD and cancer in the year 2000 was clearly less variable between countries; the probability of dying from CVD was lower than the probability of dying from cancer in 10 of 12 countries. In the NIS, there was a marked decrease in CVD premature mortality in the period 2000–19; however, the probability of CVD premature death remained five times higher compared to the selected EU countries. For cancer, the reductions in premature mortality were evident in several countries, particularly in Kazakhstan, while mortality changes were rather minimal in Azerbaijan, Republic of Moldova, Tajikistan and Turkmenistan, and even increasing in Georgia. In the EU countries—where rates were much lower than the NIS—the relative reduction in mortality was comparable for CVD and cancer. The observed trends were relatively similar for men and for women, though the decline in cancer mortality in EU-27 was more pronounced in men (Supplementary figure S2A and B).

### Will countries meet the SDG3.4 target for cancer?

**Figure 4:**
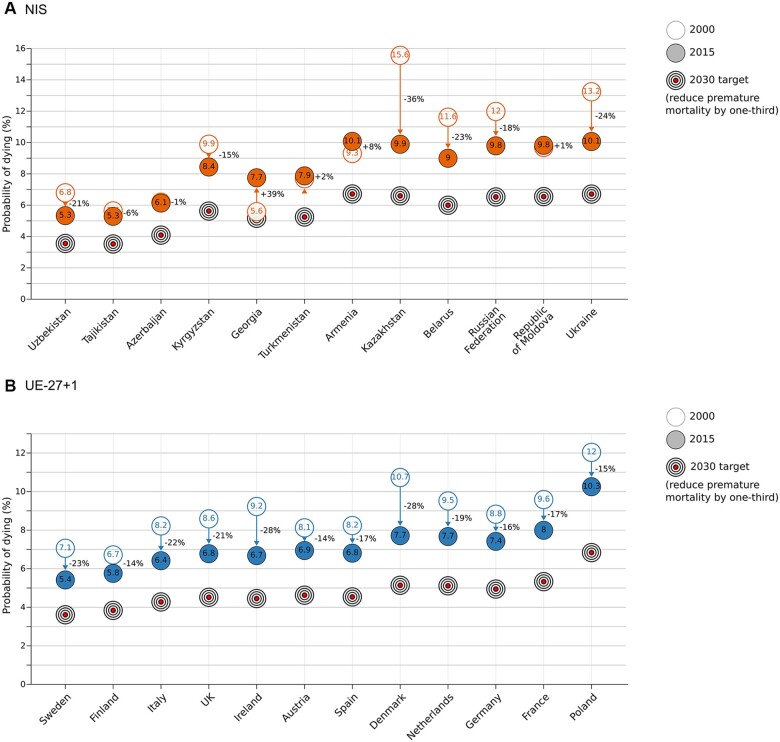
Progress in meeting SDG target 3.4 for cancer in NIS (A) vs. selected EU-27 + 1 (B) countries. EU-27 + 1, 27 EU countries and the UK; NIS, Newly Independent States of the former Soviet Union; Probability of dying, unconditional probability of dying between ages 30 and 70 years

Figure 4 and Supplementary table S1 show the absolute reduction in the probability of premature death from cancer in (A) selected NIS and (B) selected EU countries, benchmarked against the 2030 target of a one-third reduction in the 2015 probability of dying. While several EU countries are close to reaching the target, with overall reductions ranging from around 20% (France and Germany) to around 35% (Ireland and Denmark) (figure 4B), in the NIS countries, progress was mixed with reductions ranging from 2% to 8% in Azerbaijan, Republic of Moldova, Tajikistan and Turkmenistan. There were however large declines in Kyrgyzstan of 30% and particularly, Kazakhstan of 44% over the 20 years, the only two NIS on course to meet the 2030 target, in terms of cancer.

## Discussion

The results of our study confirm a divergence in the extent of epidemiological and cancer transitions across the WHO/Europe, reflecting historical geopolitical divide and contrasting health systems in specific countries, as well as policies and progress in cancer control.^10^^,^^11^ The premature cancer mortality profiles observed in our study, with a high contribution of infection-related cancers in the NIS, further confirm this EU-NIS divide.

The shift of the morbidity and mortality burden towards NCDs^12^ was followed by a decline in mortality from coronary heart disease and stroke in the second half of the 20th century.^13^^,^^14^ In Western Europe, the economic growth with improved health care (e.g. detection and treatment of hypertension, cancer screening and improved treatment) and health promotion and protection policies (e.g. tobacco control and improved road safety) have contributed to steady declines in NCD mortality and increasing life expectancy;^15^ these policies have been further extended across the EU member states, with overall EU health policy serving to complement those nationally.^16^ In contrast, the collapse of the Soviet Union in 1991, followed by an abrupt free-market transition and reduced governmental funding for health, eliminated most social-safety mechanisms for millions of people, resulting in the sharpest rise in extreme poverty.^17^ This led to a considerable loss in average life expectancy (particularly among men) in the NIS in the early-1990s, mainly attributable to increased CVD mortality, for which a subsequent peak and decline was only seen from the mid-2000s.^15^^,^^18^ While some progress in premature CVD mortality reduction has been made in the NIS in recent years, particularly in countries with the highest burden, CVD is still responsible for over 40% of deaths in the region. Five times higher premature mortality from CVD in the NIS relative to countries in the EU has been attributed to hazardous alcohol consumption and high smoking prevalence in men, as well as inadequate health services against a backdrop of greater social inequalities.^18^

A massive anti-alcohol campaign introduced in the USSR in 1985 was abandoned prior to the dissolution of the Soviet Union and was followed by increased alcohol consumption thereafter.^15^^,^^19^ Heavy episodic drinking has been identified as the main determinant of the loss of life expectancy in 1990s and a major cause of premature deaths in men.^18^^,^^20^ A recent review of alcohol control policies in 15 NIS showed different levels of alcohol consumption across the region, with decreases observed in the 2010s in countries with the highest consumption, contrasting with a rise in alcohol drinking in countries with relatively low levels of consumption, including Azerbaijan, Tajikistan and Turkmenistan.^19^

The opening of the NIS countries to international tobacco companies in the 1990s, with aggressive marketing towards women and young people increased tobacco consumption in the region.^21^ Declines in smoking prevalence, in particular in men have been reported subsequently since the 2000s,^21^ and all countries ratified or acceded to the Framework Convention for Tobacco Control.^22^

In terms of cancer, the premature mortality reduction target is well on the way to being achieved in the EU countries. In the NIS countries studied here, progress in reducing premature cancer mortality contrasts with the relative success for CVD, other than in Kazakhstan and Kyrgyzstan, which are both on the way to meeting the 2030 target for cancer. Despite comparatively lower incidence rates,^23^ the probability of premature death from cancer in the NIS is higher than in the selected EU countries, suggesting that limited health care systems and an absence of operational national cancer plans contribute to the overall lack of progress in reducing the cancer burden.^24^

A recent review examining pathways to achieve SDG target 3.4 highlighted the need for accessible and equitable health systems that have integrated population-based prevention across the continuum of care.^6^ The health care systems in the NIS have developed from a centralized state-owned Semashko system in the former Soviet Union, characterized by free provision of services and emphasis on hospital care.^18^ Today, the NIS are at different stages of a transition towards insurance-based systems, where some services are guaranteed for free while others require out-of-pocket payments.^25^ Access to treatment is variable both between and within countries, with an European Society for Medical Oncology survey reporting substantial disparities in the availability of antineoplastic medicines and the extent of out-of-pocket costs in Western vs. Eastern European countries, and in the NIS in particular. While the divide is most pronounced for new expensive non-curative chemotherapy agents, it was also apparent for therapies included in the WHO Essential Medicines List (EML). As an example, each of the 20 cancer-specific items included in the EML require full payment in Kyrgyzstan, compared to a 50% subsidy in Armenia. In Georgia and Turkmenistan, only four medications from the EML were available free of charge.^25^ Overall, Central Europe, Eastern Europe and Central Asia regions have had a consistently lower Universal Health Coverage Index than high income countries,^26^ with particular challenges in Transcaucasia and Central Asia in attaining an increase in coverage of essential health services to meet current targets.^27^

In terms of early detection, the traditions of opportunistic screening for different cancer sites in broad age groups at the level of primary care (dispensaries and/or policlinics) frequently by non-standard methods tend to be upheld across the region.^28^ Breast cancer remains the leading cause of cancer death in the NIS among women, followed by colorectal and cervical cancer. We have recently documented the later stage at diagnosis of both breast and cervical cancer in the NIS compared with EU countries, as well as the rising trend in cervical cancer incidence.^28^ An additional concern is the poor coverage of human papillomavirus vaccination in the region, other than in Turkmenistan and Uzbekistan.^29^ A quick move to evidence-based early detection practices will be essential in reducing mortality from cancers amenable to early detection; the recent launch of WHO Global Initiatives focusing on combatting cervical and breast cancers in 2021 will provide a framework for the scale-up of effective and cost-effective interventions and the monitoring of progress.^30^^,^^31^

This study has sought to assess progress towards the SDG target 3.4 for cancer in the NIS relative to selected EU 27 + 1 countries. The quality of the mortality can be considered adequate for such purposes,^32^ although a limitation is the inability to address within-country differences. Nevertheless, at the national level, we can conclude that progress in most NIS is too slow for most countries to reach the NCDs target overall and specifically for cancer. Even among those NIS with marked cancer mortality reductions, the probability of death from cancer at ages 30–70 remains higher than in the selected EU countries, signalling the need for scale-up of cancer control interventions across the region.

## Supplementary data

Supplementary data are available at *EURPUB* online.

## Funding

The contribution of A.Z. was partly supported by the WHO EURO thanks to a voluntary donation by Germany.

## Disclaimer

Where authors are identified as personnel of the International Agency for Research on Cancer/World Health Organization, the authors alone are responsible for the views expressed in this article and they do not necessarily represent the decisions, policy or views of the International Agency for Research on Cancer/World Health Organization.

*Conflicts of interest*: None declared.

Key points

- We compared the progress in cardiovascular disease (CVD) and cancer premature mortality reductions in the period 2000–19 between the Newly Independent States of the former Soviet Union (NIS) and selected European Union (EU) countries.
- The NIS achieved major reductions in premature CVD mortality, although the probabilities of death remained about five times higher in the NIS compared to the EU by 2019.
- We observed no major reductions in cancer mortality in the NIS, apart from in Kazakhstan and Kyrgyzstan.
- While the NIS appear to be on a pathway towards effective CVD control, there is a need to scale-up cancer control activities, optimally via time-bound and budgeted national cancer control plans, including a shift from opportunistic to evidence-based early detection practices.

## Data availability

Data used for the study are available from WHO Global Estimates^1^ and GLOBOCAN 2020^7^ databases.

## Supplementary Material

ckac030_Supplementary_Data
