# Supplementary material for: Progress in reducing premature mortality from cancer and cardiovascular disease in the former Soviet Union, 2000–19
Source: Eur J Public Health. 2022 Apr 20;32(4):624–9. doi: 10.1093/eurpub/ckac030 (PMC9341639; doi:10.1093/eurpub/ckac030)
Supplement: ckac030_Supplementary_Data [file ckac030_supplementary_data.zip › ejph-2021-11-om-1089-File006.docx]

**Supplementary table 1. Progress in meeting SDG target 3.4 for cancer (one-third premature mortality reduction) in selected NIS^1^ and EU-27+1^2^**

| **Country** | **Probability of death in 2000, ages 30-70** | **Probability of death in 2015, ages 30-70** | **Probability of death in 2019, ages 30-70** | **2030 target probability of death** | **Predicted year target met** |
| --- | --- | --- | --- | --- | --- |
| **NIS** |  |  |  |  |  |
| Uzbekistan | 6.8 | 5.3 | 5.1 | 3.6 | 2036 |
| Tajikistan | 5.6 | 5.3 | 5.2 | 3.5 | 2085 |
| Azerbaijan | 6.2 | 6.1 | 5.9 | 4.1 | 2158 |
| Kyrgyzstan | 9.9 | 8.4 | 6.9 | 5.6 | 2028 |
| Georgia | 5.6 | 7.8 | 7.2 | 5.2 | NA |
| Turkmenistan | 7.7 | 7.9 | 7.6 | 5.3 | 2331 |
| Armenia | 9.3 | 10.1 | 8.1 | 6.7 | 2040 |
| Kazakhstan | 15.6 | 9.9 | 8.8 | 6.6 | 2026 |
| Belarus | 11.6 | 9.0 | 8.8 | 6.0 | 2039 |
| Russian Federation | 12.0 | 9.8 | 9.1 | 6.5 | 2037 |
| Republic of Moldova | 9.7 | 9.8 | 9.4 | 6.6 | 2188 |
| Ukraine | 13.2 | 10.1 | 9.7 | 6.7 | 2036 |
| **EU 27+1** |  |  |  |  |  |
| Sweden | 7.1 | 5.4 | 4.9 | 3.6 | 2031 |
| Finland | 6.7 | 5.8 | 5.3 | 3.8 | 2038 |
| Italy | 8.2 | 6.4 | 5.9 | 4.3 | 2032 |
| UK | 8.6 | 6.8 | 6.1 | 4.5 | 2031 |
| Ireland | 9.2 | 6.7 | 6.1 | 4.5 | 2029 |
| Austria | 8.1 | 6.9 | 6.3 | 4.6 | 2036 |
| Spain | 8.2 | 6.8 | 6.3 | 4.5 | 2037 |
| Denmark | 10.7 | 7.7 | 6.7 | 5.1 | 2027 |
| Netherlands | 9.5 | 7.7 | 7.1 | 5.1 | 2035 |
| Germany | 8.8 | 7.4 | 7.1 | 4.9 | 2044 |
| France | 9.6 | 8.0 | 7.7 | 5.3 | 2043 |
| Poland | 12.0 | 10.3 | 9.5 | 6.8 | 2039 |

1 NIS: Newly Independent States of the former Soviet Union; 2 EU-27+1: 27 European Union countries and the United Kingdom
