# Supplementary figures and images for: Progress in reducing premature mortality from cancer and cardiovascular disease in the former Soviet Union, 2000–19
Source: Eur J Public Health. 2022 Apr 20;32(4):624–9. doi: 10.1093/eurpub/ckac030 (PMC9341639; doi:10.1093/eurpub/ckac030)

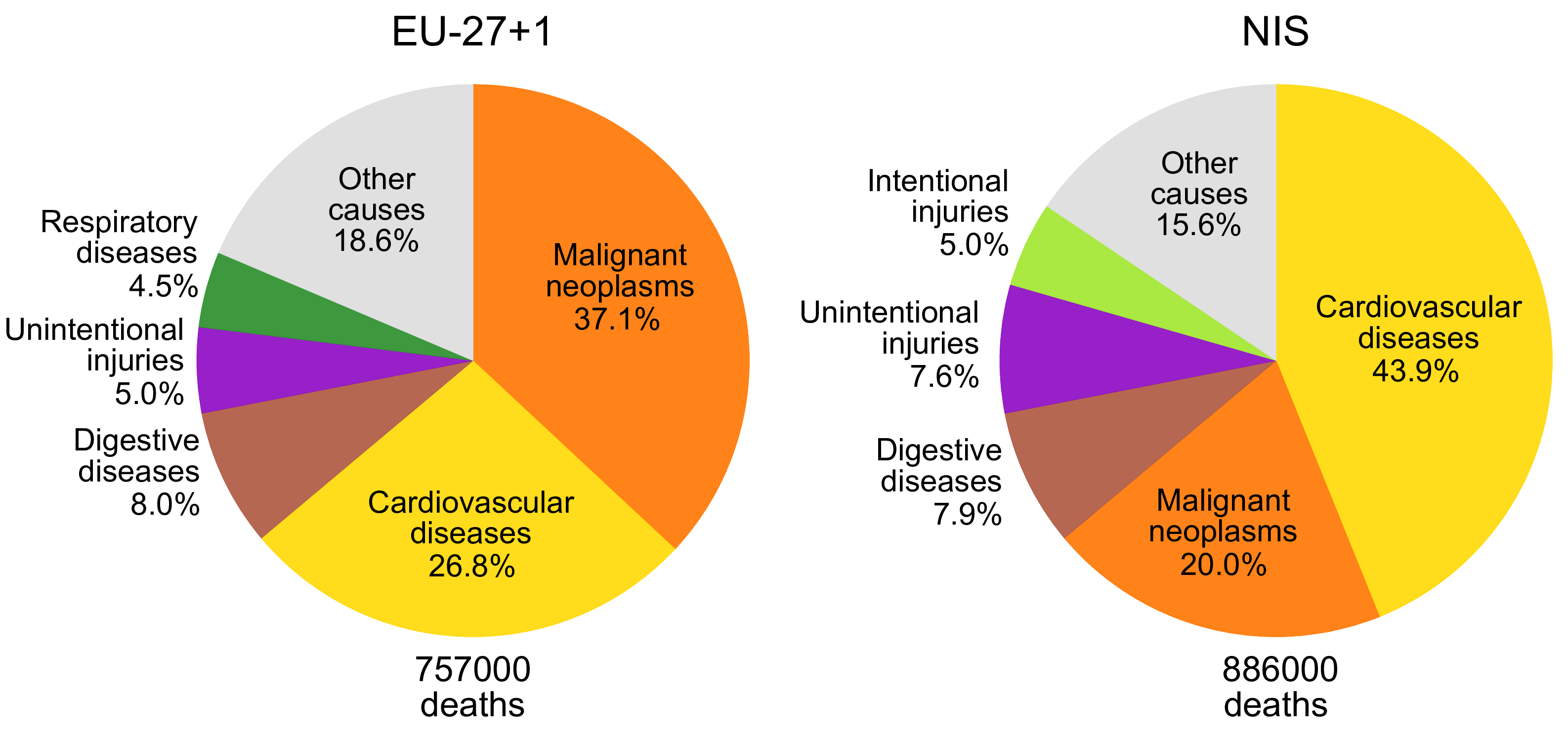

Supplement: ckac030_Supplementary_Data [file ckac030_supplementary_data.zip › ejph-2021-11-om-1089-File007.tiff]

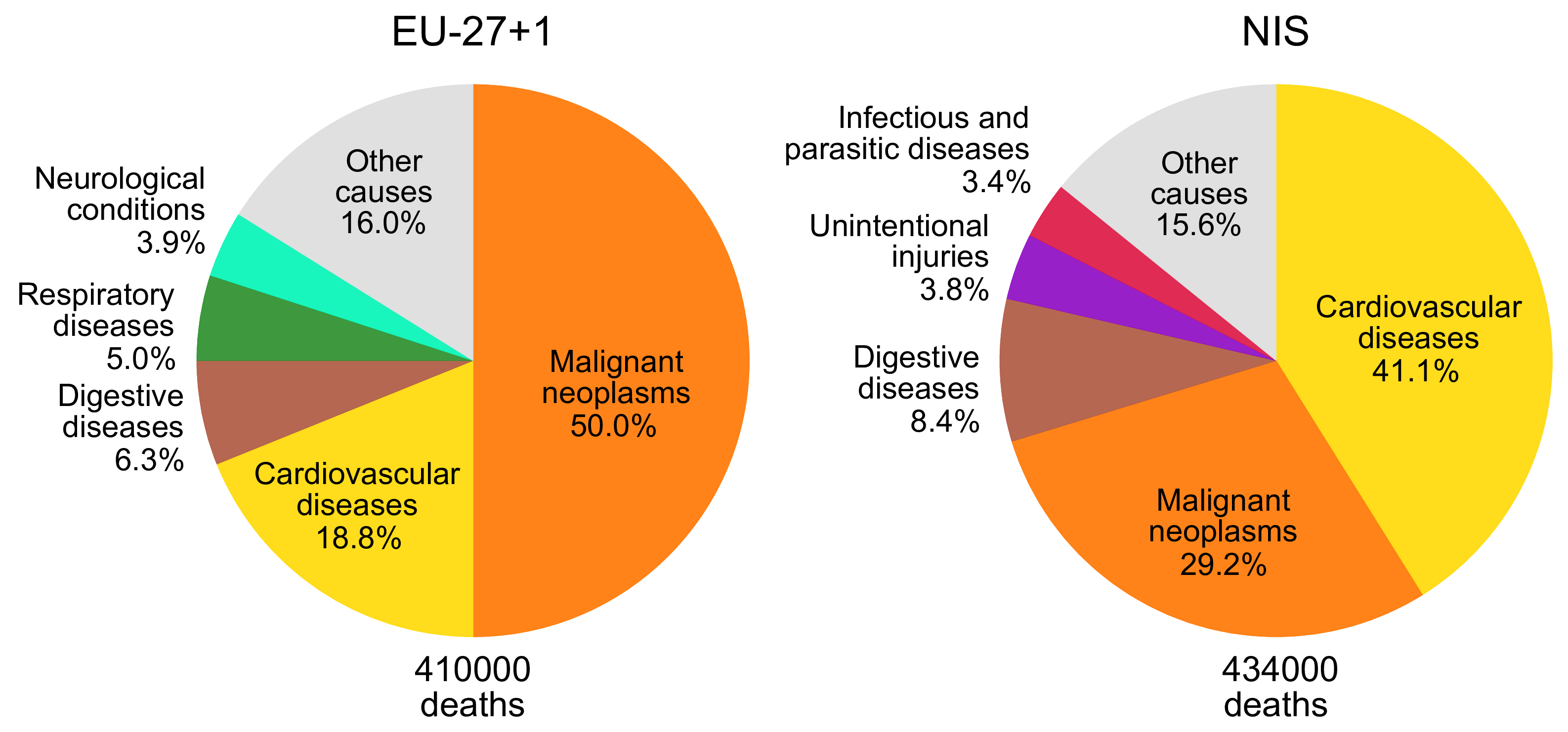

Supplement: ckac030_Supplementary_Data [file ckac030_supplementary_data.zip › ejph-2021-11-om-1089-File008.tiff]

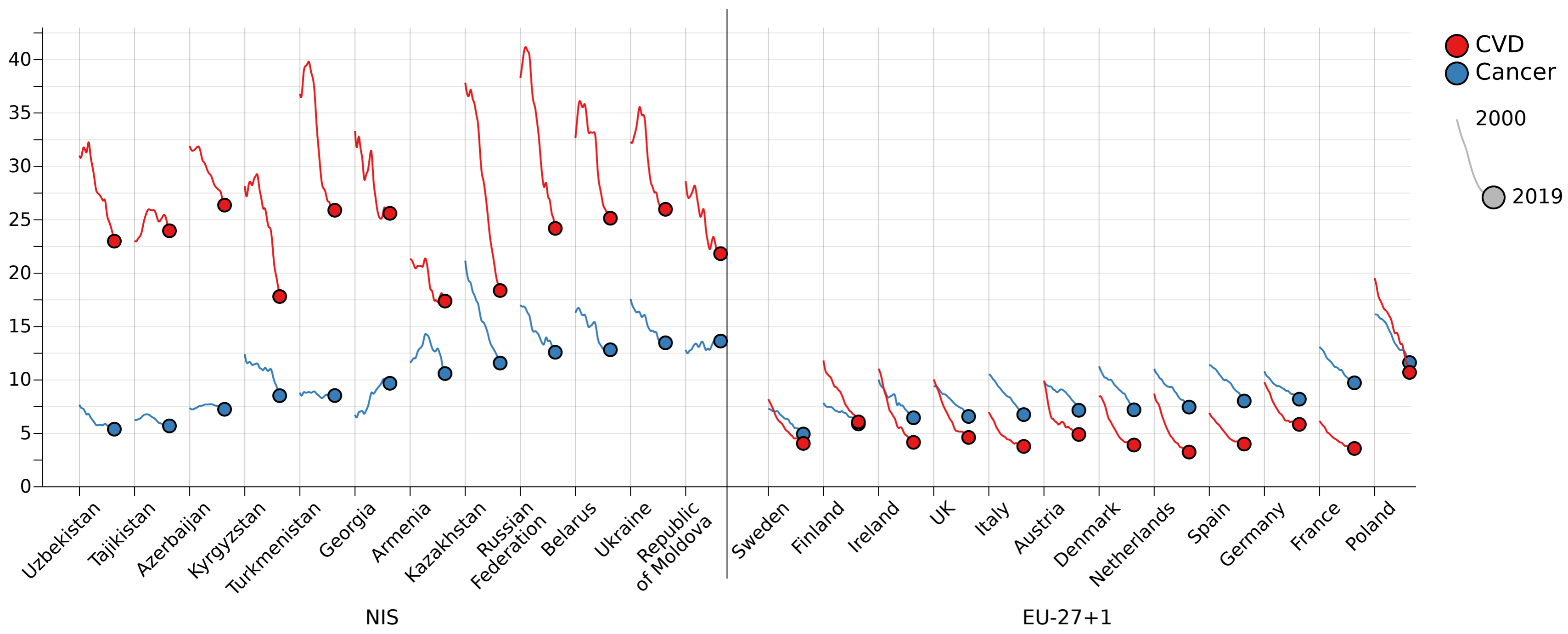

Supplement: ckac030_Supplementary_Data [file ckac030_supplementary_data.zip › ejph-2021-11-om-1089-File009.pdf]

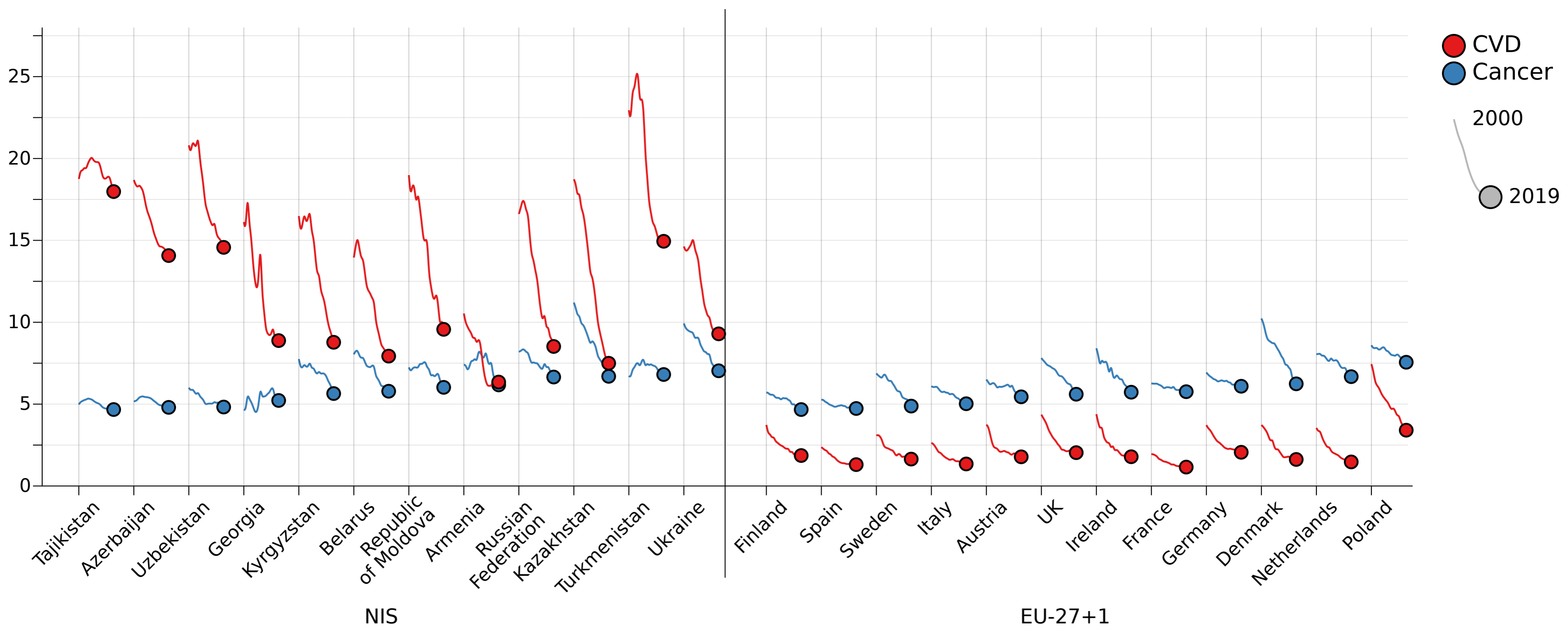

Supplement: ckac030_Supplementary_Data [file ckac030_supplementary_data.zip › ejph-2021-11-om-1089-File010.pdf]
